# A Weakly Supervised Approach for HPV Status Prediction in Oropharyngeal Carcinoma from H&E-Stained Slides

**DOI:** 10.3390/cancers17243938

**Published:** 2025-12-09

**Authors:** Angela Crispino, Silvia Varricchio, Alessandra Marfella, Dora Cerbone, Daniela Russo, Rosa Maria Di Crescenzo, Stefania Staibano, Francesco Merolla, Gennaro Ilardi

**Affiliations:** 1 Pathology Unit, Department of Advanced Biomedical Sciences, University of Naples Federico II, Via S. Pansini, 5, 80131 Napoli, Italy; angela.crispino@unina.it (A.C.); silvia.varricchio@unina.it (S.V.); alessandra.marfella@libero.it (A.M.); dora.cerbone@unina.it (D.C.); daniela.russo@unina.it (D.R.); rosamaria.dicrescenzo@unina.it (R.M.D.C.); staibano@unina.it (S.S.); gennaro.ilardi@unina.it (G.I.); 2 Department of Medicine and Health Sciences “V. Tiberio”, University of Molise, Via De Sanctis, 86100 Campobasso, Italy

**Keywords:** HPV, OPSCC, deep learning, weakly supervised learning, CLAM, whole-slide imaging, digital pathology

## Abstract

**Background:** Human papillomavirus (HPV) plays a crucial role in the pathogenesis of oropharyngeal squamous cell carcinomas (OPSCC). Accurate HPV status classification is essential for therapeutic stratification. While p16 immunohistochemistry (IHC) is the clinical surrogate marker, it has limited specificity. **Methods:** In this study, we implemented a weakly supervised deep learning approach using the Clustering-constrained Attention Multiple-Instance Learning (CLAM) framework to directly predict HPV status from hematoxylin and eosin (H&E)-stained whole-slide images (WSIs) of OPSCC. A total of 123 WSIs from two cohorts (The Cancer Genome Atlas (TCGA) cohort and OPSCC cohort from the University of Naples Federico II (OPSCC-UNINA)) were used. **Results:** Attention heatmaps revealed that the model predominantly focused on tumor-rich regions. Errors were primarily observed in slides with conflicting p16/in situ hybridization (ISH) status or suboptimal quality. Morphological analysis of high-attention patches confirmed that cellular features extracted from correctly classified slides align with HPV status, with a Random Forest classifier achieving 83% accuracy at the cell level. **Conclusions:** This work supports the feasibility of deep learning-based HPV prediction from routine H&E slides, with potential clinical implications for streamlined, cost-effective diagnostics.

## Simple Summary

Human papillomavirus (HPV) status is a key prognostic and therapeutic marker in oropharyngeal squamous cell carcinoma (OPSCC). Although p16 immunohistochemistry is widely used as a surrogate test, its limited specificity often requires additional molecular analyses, increasing diagnostic time and costs. In this study, we applied a weakly supervised deep learning (CLAM) model to predict HPV status directly from routine hematoxylin and eosin (H&E) whole-slide images, without requiring manual annotations. Using 123 slides from two independent cohorts, the model achieved good classification accuracy and consistently targeted tumor-rich regions. Importantly, in several discordant cases, the model’s predictions aligned with HPV molecular results rather than p16 staining, suggesting biological relevance. A complementary morphometric analysis at the cellular level further confirmed the distinct nuclear features associated with HPV status. Overall, this work supports the feasibility of deep learning-based HPV prediction from standard pathology slides, offering a potential complementary tool to streamline HPV testing in routine diagnosis.

## 1. Introduction

### 1.1. Human Papillomavirus (HPV) in Head and Neck Cancer

Oropharyngeal squamous cell carcinoma (OPSCC), a tumor that primarily affects the tonsils, root of the tongue, soft palate, and uvula, is undergoing one of the most significant etiological transformations [1]. Until a few decades ago, OPSCC was closely linked to known risk factors (smoking and alcohol), but today, human papillomavirus (HPV) infection has become the predominant etiological agent. This rapid transition is particularly evident in Western countries, such as North America and Northern Europe, where HPV-positive OPSCC has become the prevalent form. Here, HPV-related tumors now account for 60–80% of all OPSCC cases [1,2]. European epidemiological data confirm the extent of this change, documenting a striking three-fold increase in the incidence of HPV-positive OPSCC in males over the past twenty years [1]. Patients with HPV-positive exhibit distinct clinical features, including higher response rates to therapy, improved progression free survival, and better overall survival (OS) compared to their HPV-negative counterparts [2,3,4,5]. Given the more favorable prognosis of HPV-positive tumors and the substantial side effects associated with multimodal treatments, numerous clinical trials have investigated de-escalation treatments [6,7,8,9,10]. Consequently, accurate identification of HPV status is essential for appropriate therapeutic stratification. Currently, the standard approach relies on immunohistochemical (IHC) detection of p16, a surrogate marker for HPV, as recommended by the 8th edition of the American Joint Committee on Cancer’s (AJCC) staging system [11]. However, while p16 IHC demonstrated a high sensitivity (0.97), its specificity (0.84) remains suboptimal [12]. For this reason, p16 IHC is commonly combined with molecular assays—such as HPV DNA or RNA in situ hybridization (ISH) and PCR-based tests—to achieve a more accurate determination of HPV status [13,14]. These techniques, while useful, remain time-consuming, expensive, and not universally available. Therefore, new, complementary strategies are needed that leverage morphological information to achieve reliable HPV identification.

### 1.2. Artificial Intelligence for HPV Status Prediction from H&E Slides

Artificial intelligence (AI) and computational pathology have emerged as promising tools to address these diagnostic challenges [15,16]. Several reviews have highlighted the growing number of AI-based approaches developed for HPV status prediction in head and neck cancers, emphasizing their potential to complement or reduce reliance on expensive and time-consuming molecular testing [17,18,19,20]. In particular, deep learning models applied to hematoxylin and eosin (H&E) whole-slide images (WSIs) have demonstrated an ability to capture subtle morphological patterns associated with HPV-induced carcinogenesis, features that may be challenging to consistently recognize through human visual assessment alone. Based on this concept, Klein et al. developed a convolutional neural network (CNN)-based model that generated an HPV prediction score directly from H&E-stained slides of OPSCC, demonstrating good diagnostic performance and demonstrating that the histology-derived score stratified patient prognosis beyond conventional clinicopathological factors [21]. More recently, Wang et al. [22] proposed a novel deep learning algorithm for HPV infection prediction in a large cohort of head and neck cancers using routine histology images, reporting robust discrimination between HPV-positive and HPV-negative tumors. In our previous work, we addressed this challenge of HPV status prediction using a handcrafted machine learning approach based on single-cell morphometric features extracted from manually annotated H&E regions in OPSCC cases. The best classifier, with an accuracy above 90%, was obtained when training on cases positive both for p16^INK4a^ immunostain and for HPV DNA by ISH/INNO-LiPA^®^ [23]. This first study demonstrated that HPV infection is associated with reproducible morphological signatures observable at the single-cell level, confirming the biological plausibility of morphology-based prediction. This approach, like the more recent fully supervised models, is effective in navigating the high heterogeneity of a histological slide (which contains both tumor and non-tumor tissue) because they rely on detailed annotations that clearly isolate the tumor region of interest (ROI). However, they suffer from a crucial limitation: they rely on extensive manual annotation, which is costly in terms of time and resources. To address these limitations, we investigated a weakly supervised deep learning approach that requires only slide-level labels. To this end, we implemented Clustering-constrained Attention Multiple-Instance Learning (CLAM) [24] to directly predict HPV status from whole-slide images (WSIs) of H&E-stained OPSCC. CLAM is a well-known weakly supervised framework that uses a robust attention mechanism to identify and focus on the most predictive histological areas. This mechanism not only enables classification without explicit ROI annotation, but also provides valuable interpretability of what the model is learning, enabling the localization of relevant histological patterns. In this study, we tested the feasibility of using CLAM to directly predict HPV status from H&E-stained WSIs of OPSCC cases, and we further analyzed whether high-attention regions correspond to morphologically relevant cell types using an auxiliary feature-based classifier.

## 2. Materials and Methods

### 2.1. Data Collection

We collected H&E-stained WSIs from two distinct cohorts. The first cohort is from The Cancer Genome Atlas-Head and Neck Squamous Cell Carcinoma (TCGA-HNSC) and includes 10 WSIs of OPSCC. HPV status for these cases was determined using both p16^INK4a^ immunohistochemistry (IHC) and in situ hybridization (ISH), with five cases classified as HPV-positive and five as HPV-negative. The second cohort, named OPSCC-UNINA, includes 113 histological slides of OPSCC obtained from the archives of the Pathology Unit of the University “Federico II” of Naples. The HPV infection status of these specimens was determined by p16^INK4a^ IHC. In a subset of cases, additional molecular confirmation was available via ISH/INNO-LiPA^®^. The detailed methodology for both techniques has been previously described [23]. However, to ensure homogeneity in labeling for training the deep learning model, HPV status determined by p16 IHC was used as ground truth for all UNINA samples. Among the OPSCC-UNINA cases, 41 were HPV-positive and 72 were HPV-negative. For external validation, we used an independent set of 35 HPV-negative WSIs from UNINA, which were entirely separate from the 113 cases included in the OPSCC-UNINA training cohort. The full lists of cases analyzed in the present study are in Supplementary File S1.

### 2.2. Slide Digitization

The histological slides of the OPSCC-UNINA cohort were digitized using a Leica Aperio AT2 scanner (Leica Biosystems Imaging, Vista, CA, USA) at 20× magnification. Before scanning, each slide was carefully cleaned with solvent and sterile gauze to remove any contaminants or artifacts that could compromise image quality. This ensured optimal conditions for image analysis before model training.

### 2.3. Workflow Overview

**Figure 1:**
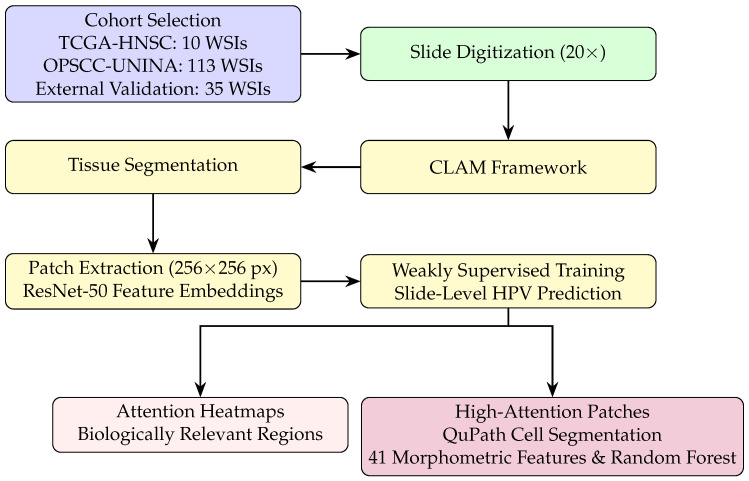
Workflow of the CLAM-based weakly supervised analysis with preprocessing, slide-level classification, and attention-guided morphometric validation.

Figure 1 summarizes the full analytical pipeline, including cohort selection, WSI preprocessing, weakly supervised learning, and attention-guided morphometric validation.

### 2.4. Computational Framework: CLAM

#### 2.4.1. Overview

To implement a weakly supervised learning approach for whole-slide image (WSI) classification, the Clustering-constrained Attention Multiple-Instance Learning (CLAM) framework [24] was adopted. This approach allows for whole-slide predictions without the need for patch- or region-of-interest (ROI)-level annotations. We used the official implementation released by the Mahmood Lab (https://github.com/mahmoodlab/CLAM (accessed on 1 October 2024)). In preprocessing, WSIs were first subjected to tissue segmentation to exclude background areas and then subdivided into non-overlapping patches of size 256 × 256 pixels. Each patch was converted into a 1024-dimensional feature vector using a ResNet-50 network pre-trained on ImageNet. During training, the model evaluates and classifies all patches, assigning each an “attention score” that determines its contribution or importance to the collective slide-level representation. This representation is computed using an attention pooling rule, which combines feature vectors by weighting them according to their attention score. CLAM also includes a supervised clustering part where the patches with the highest and lowest attention scores are separated into distinct clusters. The overall loss function combines the slide-level classification loss with a patch-level clustering loss.

#### 2.4.2. Performance Evaluation and Interpretability

Training was conducted according to a 10-fold Monte Carlo cross-validation scheme, with the dataset randomly split into 80% training, 10% validation and 10% testing for each fold, maintaining the stratification by class. Optimization was performed using the Adam algorithm with a learning rate of $2\times 10^{-4}$ and an early stop criterion after 20 consecutive epochs without improvement of the validation loss, up to a maximum of 200 epochs. A batch size of 1 was used (one slide per batch) and no data augmentation techniques were applied. At the end of each fold, the model performance was evaluated in terms of accuracy (ACC) and area under the ROC curve (AUC), both on the validation and test set, using the official scripts provided by the CLAM framework. Finally, to increase the interpretability of the model, attention heatmaps were generated, highlighting the regions of the slide that most influenced the predictive decision.

### 2.5. Feature Evaluation

To assess whether high-attention regions reflected morphologically significant differences, we conducted a supervised cellular-level analysis on truly positive and truly negative patches identified by the CLAM model. A total of 1230 high-attention patches were selected and analyzed using QuPath (v0.6.0-rc3) [25]. Each patch inherited the slide-level HPV label, and cells were detected using QuPath’s cell detection module with adjusted parameters. A total of 133,125 cells were segmented and characterized by 41 morphological and staining-related features.

#### 2.5.1. Morphological Feature-Based Classification

We trained a Random Forest classifier using the extracted features to distinguish HPV-positive from HPV-negative cells. The model, implemented in Python 3.10 using the scikit-learn library, consisted of 500 trees and default hyperparameters. The analysis was conducted following a formal approach, previously described and validated by Varricchio et al. [23]. Source code was adapted from the official scikit-learn documentation https://scikit-learn.org/stable/modules/generated/sklearn.ensemble.RandomForestClassifier.html (accessed on 1 October 2024).

#### 2.5.2. Computational Environment

All experiments were conducted on a Windows workstation equipped with an NVIDIA RTX A2000 GPU (12 GB VRAM), Intel(R) Xeon(R) W5-3465X CPU, and 64 GB of RAM.

## 3. Results

### 3.1. CLAM Model Performance on Internal Cross-Validation

We trained a CLAM model on 123 Whole-Slide Images (WSIs) from two cohorts: OPSCC-UNINA (n = 113) and TCGA (n = 10). In the 10-fold cross-validation setting, the CLAM model showed moderate performance with substantial variability across folds (Table 1). The average test area under the curve (AUC) was 0.5324, and the corresponding test accuracy was 56.5%, whereas validation performance was consistently higher, with an average AUC of 0.7178 and accuracy of 78.2%. Notably, Fold 6 achieved a perfect test AUC of 1.0 and the highest test accuracy of 80.0%, suggesting optimal class separation within that subset (Table 1).

### 3.2. Global Classification and Probability Analysis

**Figure 2:**
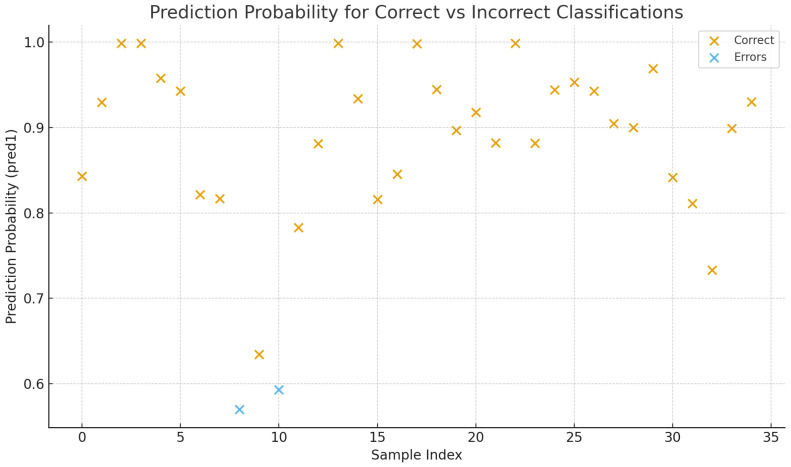
Scatter plot showing the model’s prediction probabilities for the external test set. Each point represents one tile from the 35 HPV-negative WSIs. Orange markers indicate tiles correctly classified as HPV-negative, while blue markers highlight misclassified tiles predicted as HPV-positive. Misclassified samples display borderline confidence values (0.593 and 0.570), consistent with predictions near the decision threshold. These tiles originated from a slide containing a large air bubble.

For each WSI, the model outputs both the predicted class and the associated probabilities for HPV-positive and HPV-negative labels. Overall, 78.9% of WSIs were correctly classified (Table 2), with the majority showing high confidence. However, about 21% of the WSIs (26 out of 123, of which 23 from the OPSCC-UNINA dataset and 3 from TCGA) were misclassified (Figure 2) (Supplementary File S2).

Among the 21% of misclassified WSIs (26 of 123), several recurring patterns were identified. In four WSIs, the model prediction probabilities were close to the classification threshold, suggesting uncertainty in the decision-making process. These borderline cases often showed nearly equal probabilities for both HPV-positive and HPV-negative classes (e.g., 0.512 vs. 0.487). In four other WSIs, model predictions were discordant with IHC-based labels but concordant with INNO-lipa results. Some samples labeled as HPV-positive by IHC but negative by INNO-lipa were also predicted as negative by the model. This observation suggests that p16 IHC, while commonly used as a surrogate marker, may occasionally lead to false-positive classifications. These findings emphasize the added value of computational models in flagging cases that are ambiguous or potentially misclassified when relying on a single biomarker. Six additional WSIs had significant artifacts, such as tissue folds, tears, or glass markers, which likely impaired the model’s ability to extract relevant histological features. Notably, all three misclassified slides from the TCGA cohort fell into this category, indicating a potential link between technical quality and misclassification.

### 3.3. External Test Set Performance

The trained model was evaluated on an independent test set of 35 HPV-negative WSIs. The model correctly classified 33 out of 35 cases, yielding an accuracy of 94.3%. Most correctly classified slides had strong negative probabilities (80–90%). The two misclassified slides were predicted as HPV-positive with probabilities of 0.593 and 0.570, both of which are close to the classification threshold. One of these slides contained a large air bubble, likely compromising the feature extraction process (Supplementary File S3).

### 3.4. Interpretability Through Attention Maps

**Figure 3:**
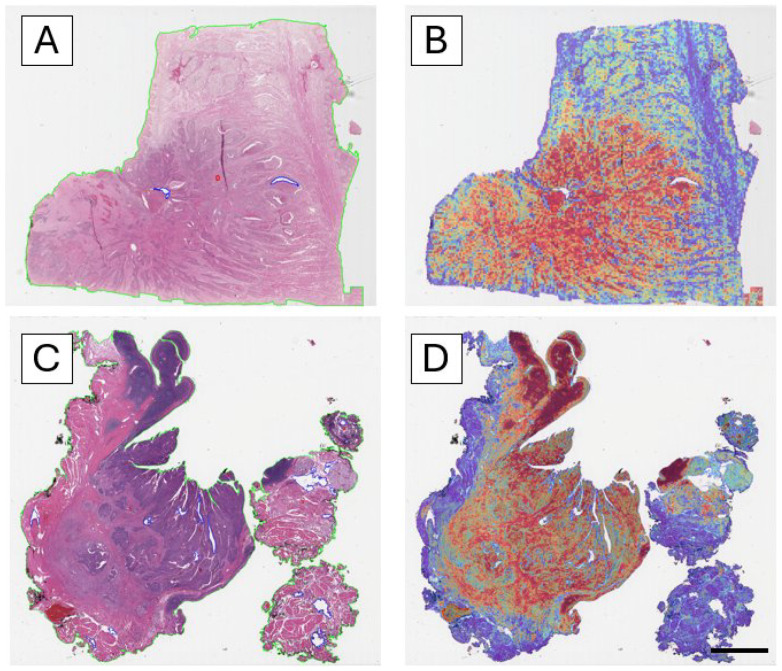
**Interpretability of the model through attention heatmaps.** Attention maps use a color scale ranging from **blue** (low attention/probability) to **red** (high attention/probability), where red highlights the regions that most strongly contribute to the model’s prediction. (**A**) H&E whole-slide image of a correctly classified HPV-negative case. (**B**) Corresponding attention heatmap showing high-attention areas (red–yellow) concentrated in tumor-rich regions. (**C**) H&E whole-slide image of a correctly classified HPV-positive case. (**D**) Corresponding attention heatmap, again highlighting strong attention over tumor regions. Across both negative and positive cases, the model consistently focuses on tumor-rich regions, indicating that it learns morphologically relevant patterns for HPV status prediction (scale bar: 50 pixel size).

Analysis of the attention heatmaps revealed that the model consistently focused on tumor-rich regions, particularly in correctly classified cases (Figure 3). This behavior was observed across both training and external datasets, supporting the hypothesis that the model learns biologically relevant features for HPV status classification.

### 3.5. Cell-Level Analysis

**Figure 4:**
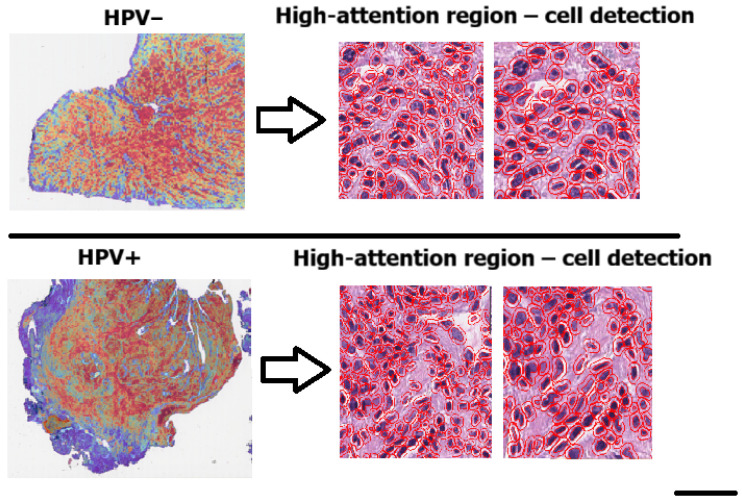
Cell detection performed with QuPath within the high-attention patches (red regions in the CLAM heatmaps). These regions were used to extract single-cell morphometric features for the Random Forest analysis (scale bar: 50 pixel size).

**Figure 5:**
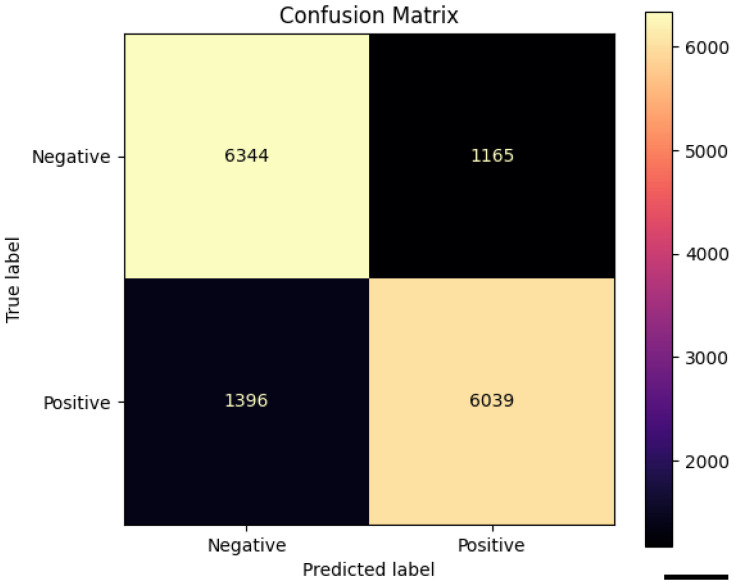
Confusion matrix of the Random Forest classifier trained on 41 cell-level morphometric features extracted from high-attention regions selected by CLAM. The model shows comparable performance across classes (6344 true negatives and 6039 true positives), supporting the presence of consistent cellular-level morphological signatures driving the weakly supervised model’s predictions (scale bar: 50 pixel size).

To further evaluate whether the CLAM model was focusing on biologically meaningful regions, we performed a cell-level morphometric analysis restricted to the high-attention patches. Specifically, we extracted only the patches corresponding to the highest-attention areas (red regions in the CLAM heatmaps) and applied QuPath v0.6.0 to perform cell detection within these regions. For each detected cell, QuPath provided 41 morphological and staining-related features. Each cell inherited the HPV label of the high-attention patch from which it was extracted. Figure 4 We trained a Random Forest classifier on a balanced dataset of 74,718 cells (37,359 per class), each labeled according to the patch from which it was extracted. The model achieved an overall accuracy of 82.9%, with a precision of 84%, recall of 81%, and F1-score of 0.83 for the HPV-positive class, as summarized in Table 3. Classification performance was consistent across both classes, confirming the presence of robust morphometric signals differentiating HPV-related tumors. The confusion matrix shown in Figure 5 further confirms the balanced performance of the classifier, with comparable accuracy in both HPV-positive and HPV-negative classes.

## 4. Discussion

Predicting HPV status directly from H&E-stained slides using deep learning represents a promising and innovative frontier in computational pathology. Building on our previous work with handcrafted morphological features, which demonstrated high accuracy in distinguishing HPV-positive from HPV-negative OPSCC and highlighted the complementary nature of p16 IHC and molecular testing, we sought to explore a more scalable, annotation-free approach. In this study, we employed the CLAM framework, a weakly supervised deep learning model, to classify HPV status using only slide-level labels. The model achieved an overall classification accuracy of 78.9% on the internal dataset and performed strongly on an independent test set, reaching 94.3% accuracy. These findings confirm the feasibility of extracting biologically relevant features from routine H&E slides without manual region-of-interest annotation.

The attention maps generated by the CLAM model consistently highlighted tumor-rich epithelial regions, indicating that these areas contributed most to the slide-level HPV prediction. High-attention zones corresponded to morphologically distinctive regions, often characterized by cohesive tumor nests with atypical nuclei and reduced keratinization—features typically associated with HPV-related OPSCC. This spatial localization suggests that the model prioritizes biologically relevant structures rather than stromal or artefactual components, providing a visual representation of the regions driving the classification.

Despite these encouraging results, performance during cross-validation was variable (mean AUC: 0.5324), with one fold achieving perfect discrimination (AUC = 1.0; accuracy = 80%). More specifically, the discrepancy between validation and test performance (mean validation AUC: 0.7178 vs. mean test AUC: 0.5324) and the wide range of test AUC values across folds (0.18–1.00, Table 1). This variability is likely due to the limited number of slides per fold, the intrinsic heterogeneity of OPSCC, and occasional image artefacts, all of which may have affected model performance.

Moreover, our cross-validation strategy relied on random slide-level splits, which may have further amplified these fluctuations. Label noise may also have contributed. HPV status was assigned mainly by p16 IHC; therefore, occasional p16-positive but truly HPV-negative tumours may have led to mislabelled cases, increasing performance variability. In this context, the very high AUC observed in fold 6 most likely reflects a particularly favourable distribution of clearly positive and clearly negative cases rather than a genuinely superior model. Overall, these findings highlight the need for larger, multi-institutional cohorts and more specific and harmonised ground-truth definitions to obtain more stable estimates of weakly supervised model performance. Recent studies, such as Klein et al. (AUC = 0.80) [26], Wang et al. (AUC = 0.8371) [22], and Adachi et al. (AUC = 0.905) [27], performed better. Comparative tables with previously published works are in Supplementary File S4. However, direct comparison with these benchmark studies is not straightforward, as they differ substantially in cohort size, population structure, and evaluation strategy. While these studies often rely on larger or more homogeneous datasets and standardized workflows, our contribution is to demonstrate that weak supervision remains effective even in the absence of manual annotations, producing interpretable attention maps and identifying cases that may be misclassified by p16 IHC under real-world conditions.

Indeed, in four misclassified cases, model predictions were discordant with p16 status but aligned with the INNO-LiPA results, suggesting that the model may detect histological cues more closely associated with actual HPV infection. Such findings reinforce the notion that computational models can serve as a second opinion or flag potentially misclassified cases when relying solely on p16. Technical artifacts also contributed to misclassification. Slides with tissue folds, air bubbles, or annotation markers often impaired model performance, underscoring the need for quality control as an integral component of digital pathology workflows. To enhance model interpretability, attention heatmaps were generated, which consistently localized to tumor-rich areas.

**Figure 6:**
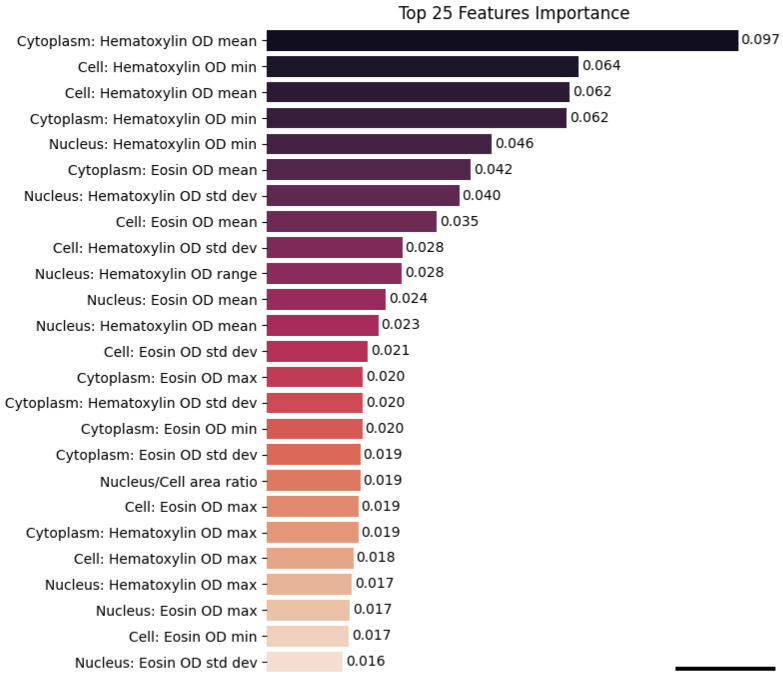
Feature importance ranking from the Random Forest classifier trained on 41 cell-level morphometric and staining-related features. The plot displays the top 25 most important features, highlighting their relative contribution to the classification of HPV-positive and HPV-negative cells. Notably, haematoxylin-related nuclear metrics emerge as the most influential predictors (scale bar: 50 pixel size).

To further investigate the nature of these regions, we extracted 1230 high-attention patches and performed supervised cell-level morphological analysis using QuPath. The Random Forest classifier trained on the resulting 133,000 segmented cells achieved an accuracy of 82.9%. Feature importance analysis revealed that the most influential variables were colorimetric, particularly haematoxylin optical density metrics associated with nuclear morphology and chromatin texture (Figure 6). Remarkably, these same features emerged as top contributors in our prior feature-based machine learning model, which was trained on manually annotated cells, suggesting strong biological consistency between traditional and deep learning approaches [23].

Nuclear chromatin structure and hematoxylin optical density, the morphometric characteristics that our research found to be the most discriminative, are very consistent with the known biological distinctions between HPV-positive and HPV-negative OPSCC [28,29,30,31]. The fact that these biologically grounded nuclear features were consistently ranked as the most important predictors in the Random Forest model—and were concentrated within the high-attention CLAM regions—supports the conclusion that the weakly supervised classifier is detecting true HPV-associated nuclear phenotypes rather than spurious visual patterns. This congruence between known disease abnormalities and computational features increases the model’s interpretability in a clinical setting and supports the biological plausibility of its predictions.

Overall, our findings support the potential of weakly supervised deep learning to deliver both accurate and interpretable predictions of HPV status in OPSCC. The reproducibility of key morphometric features, alignment with molecular results in ambiguous cases, and localization of model attention to biologically plausible regions are all hallmarks of a robust pipeline.

The introduction of weakly supervised deep learning models into HPV diagnostic workflows also requires careful recalibration of diagnostic cut-off thresholds. Traditional thresholds optimized for p16 IHC or molecular assays may not fully capture the confidence distributions generated by AI-based classifiers. Establishing method-specific and biologically informed cut-offs will therefore be essential to ensure reliable integration of these models into routine OPSCC diagnostics [32,33]. For clinical adoption, however, seamless integration into laboratory workflows remains essential. The recent framework proposed by Angeloni et al. [34], which enables HL7-based communication between AI systems and laboratory information systems (LIS), represents a critical step toward deployment. Equally important is integrating predictive outputs—such as heatmaps or confidence scores—into widely used platforms like QuPath to support daily diagnostic utility.

## 5. Limitations and Future Directions

The main limitation of the present study is that the cohort size (123 WSI), although comparable to previous reports, remains relatively small and contributed to cross-validation variability. Moreover, ground-truth definition was largely based on p16 IHC, which, while widely used, may misclassify some HPV-negative tumours as positive, introducing label noise that affects model stability. External validation was limited to HPV-negative cases, preventing a full assessment of generalizability. Despite these limitations, the model produced biologically plausible attention maps and, in discordant cases, its predictions aligned with molecular INNO-LiPA results rather than p16, suggesting that weakly supervised approaches can capture true HPV-related morphology. Future work will include expanding to a multi-institutional dataset, applying stricter quality control to mitigate artefacts, and refining ground-truth labels by integrating molecular HPV detection alongside p16.

## 6. Conclusions

In summary, we demonstrate that weakly supervised deep learning can predict HPV status directly from routine H&E-stained OPSCC slides using only slide-level labels. Although cross-validation performance was mixed, the model achieved good overall accuracy on the internal cohort and high specificity on an independent set of HPV-negative tests. Attention heatmaps consistently highlighted tumor-rich regions, and a complementary cellular-level analysis confirmed that high-attention areas exhibit reproducible morphometric signatures that differ between HPV-positive and HPV-negative tumors. These results suggest that weakly supervised models such as CLAM can recover biologically meaningful patterns without manual region-of-interest annotation and could potentially complement p16 IHC and molecular testing.

## Figures and Tables

**Table 1:** Performance metrics of the CLAM model across the 10-fold cross-validation. For each fold, the table reports the test and validation AUC, along with the corresponding test and validation accuracy values. The last row summarizes the average performance across all folds.

Fold	Test AUC	Val AUC	Test Acc	Val Acc
0	0.4821	0.2424	0.4667	0.7857
1	0.4571	1.0000	0.4167	0.9091
2	0.7407	0.5833	0.5000	0.4000
3	0.7143	0.1750	0.6667	0.7143
4	0.5000	0.8571	0.5455	0.8889
5	0.4286	0.9394	0.6667	0.8571
6	1.0000	0.6667	0.8000	0.6429
7	0.1875	1.0000	0.5625	0.9231
8	0.4688	0.8393	0.5833	0.8000
9	0.3500	0.8750	0.4444	0.9000
Average	0.5324	0.7178	0.5652	0.7821

**Table 2:** Summary of classification results on the two datasets. The table reports, for each cohort, the total number of WSIs, the number correctly classified by the CLAM model, the number of misclassified slides, and the corresponding classification accuracy. The final row provides the aggregated results across both datasets.

Dataset	Total WSIs	Correctly Classified	Misclassified	Accuracy (%)
OPSCC-UNINA	113	90	23	79.65
TCGA	10	7	3	70.0
Total	123	97	26	78.9

**Table 3:** Classification performance of the Random Forest model at cell level. Summary of the classification metrics computed for the Random Forest model. The table reports precision, recall, and F1-score for each class (HPV-negative and HPV-positive), as well as macro and weighted averages. The overall accuracy of the model is indicated below the table.

Class	Precision	Recall	F1-Score
Negative	0.82	0.84	0.83
Positive	0.84	0.81	0.83
Macro average	0.83	0.83	0.83
Weighted average	0.83	0.83	0.83

**Accuracy: 0.83.**

## Data Availability

The data used in this study are available partly from public repositories and partly from institutional archives. The TCGA-HNSC whole-slide images and associated metadata are publicly accessible via the GDC Data Portal: https://portal.gdc.cancer.gov/projects/TCGA-HNSC (accessed on 1 March 2025). Data from the OPSCC-UNINA cohort contain potentially identifiable patient information and are therefore not publicly available due to privacy and ethical restrictions; full case lists are provided in Supplementary File S1. The CLAM framework source code is openly available at https://github.com/mahmoodlab/CLAM (accessed on 1 March 2025). The Random Forest implementation was adapted from the official scikit-learn documentation.

## Supplementary Materials

The following supporting information can be downloaded at: https://www.mdpi.com/article/10.3390/cancers17243938/s1, Supplementary File S1—Table S1: Summary of patient characteristics and biomarker results across datasets; Supplementary File S2—Table S2: Case-level predictions and discordant results from CLAM and Random Forest models; Supplementary File S3—Table S3: Slide-level predictions with class probabilities and notes; Supplementary File S4—Table S4: Comparison of model strategies across the four studies. Table S5: Dataset characteristics across the four studies. Table S6: Ground truth definitions used in the four studies. Table S7: Model performance comparison across all studies. Table S8: Summary of strengths and limitations.

## Abbreviations

ACC	Accuracy
AJCC	American Joint Committee on Cancer
AUC	Area under the ROC curve
AI	Artificial intelligence
CLAM	Clustering-constrained Attention Multiple-Instance Learning
DL	Deep learning
FFPE	Formalin-fixed paraffin-embedded
H&E	Hematoxylin and eosin
HPV	Human papillomavirus
IHC	Immunohistochemistry
ISH	In situ hybridization
ML	Machine learning
OPSCC	Oropharyngeal squamous cell carcinoma
OS	Overall survival
ROI	Region of interest
RF	Random Forest
TCGA	The Cancer Genome Atlas
WSI	Whole-slide image
