# Supplementary material for: A Weakly Supervised Approach for HPV Status Prediction in Oropharyngeal Carcinoma from H&E-Stained Slides
Source: Cancers (Basel). 2025 Dec 9;17(24):3938. doi: 10.3390/cancers17243938 (PMC12730384; doi:10.3390/cancers17243938)
Supplement: Supplementary file 1 [file cancers-17-03938-s001.zip › Supplementary File S1.pdf]

Supplementary File S1

Table S1: Summary of patient characteristics and biomarker results across datasets.

**Dataset**	**Year of Diagnosis**	**Total cases**	**Age Range**	**Male**	**Female**	**p16 Neg**	**p16 Pos**	**INNOLIPA Pos**
TCGA cases	2009–2013	10	40–59	9	1	5	5	10
UNINA-OPSCC	2017–2022	113	17–87	83	30	72	41	10
External test set	2017–2022	35	23–80	nd	nd	35	–	nd
**Total**	2009–2022	**158**	17–87	**92**	**31**	**112**	**46**	**20**
