# Supplementary material for: A Weakly Supervised Approach for HPV Status Prediction in Oropharyngeal Carcinoma from H&E-Stained Slides
Source: Cancers (Basel). 2025 Dec 9;17(24):3938. doi: 10.3390/cancers17243938 (PMC12730384; doi:10.3390/cancers17243938)
Supplement: Supplementary file 1 [file cancers-17-03938-s001.zip › Supplementary File S2.pdf]

Supplementary File S2

Table S2: Case-level predictions and discordant results.

Case ID	True Label	Pred_0	p_0	Pred_1	p_1	Discordanti
1	neg	pos	0.6125	neg	0.3875	nn
2	neg	pos	0.6400	neg	0.3600	nn
3	neg	neg	0.8975	pos	0.1025	
4	neg	neg	0.9929	pos	0.0071	
5	neg	neg	0.8957	pos	0.1043	
6	neg	neg	0.8257	pos	0.1743	
7	neg	neg	0.9056	pos	0.0944	
8	neg	neg	0.9545	pos	0.0455	
9	neg	neg	0.9583	pos	0.0417	
10	neg	neg	0.9582	pos	0.0418	
11	neg	neg	0.9902	pos	0.0098	nn
12	neg	neg	0.9193	pos	0.0807	nn
13	neg	neg	0.9193	pos	0.0807	
14	neg	neg	0.9063	pos	0.0937	
15	neg	neg	0.9500	pos	0.0500	
16	neg	neg	0.9956	pos	0.0044	
17	neg	neg	0.9937	pos	0.0063	
18	neg	neg	0.9893	pos	0.0107	
19	neg	neg	0.9418	pos	0.0582	

Case ID	True Label	Pred_0	p_0	Pred_1	p_1	Discordanti
20	neg	neg	0.6389	pos	0.3611	
21	neg	neg	0.5797	pos	0.4203	
22	neg	neg	0.8720	pos	0.1280	
23	neg	neg	0.8647	pos	0.1353	
24	neg	neg	0.9396	pos	0.0604	
25	neg	neg	0.9439	pos	0.0561	
26	neg	neg	0.9440	pos	0.0560	
27	neg	neg	0.9248	pos	0.0752	
28	neg	neg	0.9202	pos	0.0798	
29	neg	neg	0.9291	pos	0.0709	
30	neg	neg	0.8191	pos	0.1810	
31	neg	neg	0.9046	pos	0.0954	
32	neg	neg	0.7872	pos	0.2128	
33	neg	neg	0.9446	pos	0.0554	
34	neg	neg	0.9331	pos	0.0669	
35	neg	neg	0.9171	pos	0.0829	
36	neg	neg	0.8398	pos	0.1602	
37	neg	neg	0.9134	pos	0.0866	
38	neg	neg	0.9426	pos	0.0574	
39	neg	neg	0.8333	pos	0.1667	
40	neg	neg	0.9864	pos	0.0136	
41	neg	neg	0.9853	pos	0.0146	
42	neg	neg	0.8919	pos	0.1080	
43	neg	neg	0.9215	pos	0.0784	
44	neg	neg	0.9114	pos	0.0885	
45	neg	neg	0.9270	pos	0.0729	
46	neg	neg	0.9403	pos	0.0596	
47	neg	neg	0.9072739	pos	0.09272612	
48	neg	neg	0.9307084	pos	0.0692916	
49	neg	neg	0.98508495	pos	0.014915106	
50	neg	neg	0.98991454	pos	0.01008552	

Case ID	True Label	Pred_0	p_0	Pred_1	p_1	Discordanti
51	neg	neg	0.98535246	pos	0.014647597	
52	neg	neg	0.98611206	pos	0.013887894	
53	neg	neg	0.96212125	pos	0.037878755	
54	neg	neg	0.9982857	pos	0.0017142883	
55	neg	neg	0.81949	pos	0.18051	
56	neg	neg	0.9977793	pos	0.0022206942	
57	neg	neg	0.98900455	pos	0.010995475	
58	neg	neg	0.933283	pos	0.06671705	
59	neg	pos	0.7601607	neg	0.2398393	artifacts
60	neg	neg	0.66795474	pos	0.33204523	
61	neg	neg	0.9960251	pos	0.003974921	
62	neg	neg	0.9984175	pos	0.0015825093	
63	neg	neg	0.99836797	pos	0.00163199	
64	neg	neg	0.9779398	pos	0.022060212	
65	neg	neg	0.9985166	pos	0.0014833374	
66	neg	neg	0.9984249	pos	0.0015751551	
67	neg	neg	0.8406594	pos	0.15934065	
68	neg	neg	0.90363	pos	0.09636995	
69	neg	neg	0.8555804	pos	0.14441963	
70	neg	neg	0.951876	pos	0.048124027	
71	neg	neg	0.7463562	pos	0.2536438	
72	neg	pos	0.52540976	neg	0.47459027	threshold close
73	pos	neg	0.8342518	pos	0.16574816	nn
74	pos	pos	0.8369978	neg	0.16300222	
75	pos	pos	0.71195483	neg	0.28804514	
76	pos	pos	0.83808196	neg	0.161918	
77	pos	neg	0.5934919	pos	0.40650815	nn
78	pos	neg	0.88339376	pos	0.1166063	artifacts
79	pos	pos	0.9453517	neg	0.05464825	
80	pos	pos	0.9494628	neg	0.05053725	
81	pos	pos	0.94189394	neg	0.05810605	

Case ID	True Label	Pred_0	p_0	Pred_1	p_1	Discordanti
82	pos	pos	0.94189394	neg	0.05810605	
83	pos	pos	0.93501836	neg	0.06498164	
84	pos	pos	0.8018591	neg	0.19814089	
85	pos	neg	0.603225	pos	0.396775	nn
86	pos	pos	0.5587886	neg	0.44121137	
87	pos	neg	0.7426036	pos	0.25739634	nn
88	pos	pos	0.92613894	neg	0.07386112	
89	pos	pos	0.7685441	neg	0.2314559	
90	pos	pos	0.88721865	neg	0.11278129	
91	pos	pos	0.92789346	neg	0.07210649	
92	pos	pos	0.9238777	neg	0.076122284	
93	pos	pos	0.5759339	neg	0.4240661	
94	pos	pos	0.81377757	neg	0.18622243	
95	pos	neg	0.731209	pos	0.26879105	pos IHC / NEG INNOipa
96	pos	pos	0.6731072	neg	0.32689282	
97	pos	neg	0.6782068	pos	0.32179326	pos IHC / NEG INNOipa
98	pos	neg	0.6779278	pos	0.32207224	nn
99	pos	neg	0.5127961	pos	0.48720387	threshold close
100	pos	neg	0.76419944	pos	0.23580052	nn
101	pos	neg	0.8497538	pos	0.15024617	nn
102	pos	neg	0.78464115	pos	0.21535882	pos IHC / NEG INNOipa
103	pos	neg	0.82106394	pos	0.17893611	pos IHC / NEG INNOipa
104	pos	pos	0.5891135	neg	0.41088656	
105	pos	neg	0.6322354	pos	0.36776465	nn
106	pos	neg	0.5587251	pos	0.44127494	threshold close
107	pos	pos	0.774931	neg	0.22506894	
108	pos	pos	0.5984151	neg	0.4015849	
109	pos	neg	0.82763034	pos	0.17236963	pos IHC / NEG INNOipa
110	pos	neg	0.97823995	pos	0.021760097	pos IHC / NEG INNOipa
111	pos	pos	0.6635037	neg	0.3364963	
112	pos	neg	0.84355426	pos	0.15644576	artifacts

Case ID	True Label	Pred_0	p_0	Pred_1	p_1	Discordanti
113	pos	neg	0.5796637	pos	0.42033625	threshold close
TCGA1	neg	pos	0.70651406	neg	0.29348597	artifacts
TCGA2	neg	neg	0.95757085	pos	0.04242916	
TCGA3	neg	pos	0.80493610	neg	0.19506383	artifacts
TCGA4	neg	neg	0.75996220	pos	0.24003781	
TCGA5	neg	neg	0.97184090	pos	0.02815908	
TCGA6	pos	pos	0.96980570	neg	0.03019426	
TCGA7	pos	neg	0.99806720	pos	0.00193282	artifacts
TCGA8	pos	pos	0.62656600	neg	0.37343400	
TCGA9	pos	pos	0.51334316	neg	0.48665684	
TCGA10	pos	pos	0.81634694	neg	0.18365304	
