# Supplementary material for: A Weakly Supervised Approach for HPV Status Prediction in Oropharyngeal Carcinoma from H&E-Stained Slides
Source: Cancers (Basel). 2025 Dec 9;17(24):3938. doi: 10.3390/cancers17243938 (PMC12730384; doi:10.3390/cancers17243938)
Supplement: Supplementary file 1 [file cancers-17-03938-s001.zip › Supplementary File S3.pdf]

Supplementary File S3

Table S3: **Slide-level predictions with class probabilities and notes.** Predictions on the external test set of 35 HPV-negative whole slide images (WSIs). For each case, the table reports the original label, the model's predicted label, and the associated prediction probabilities. Correct classifications show high confidence for the negative class, whereas the two misclassified slides display borderline positive probabilities (0.593 and 0.570). One misclassified case contained a large air bubble artifact, likely affecting feature extraction and contributing to the incorrect prediction.

#	original	pred1	prob1	pred2	prob2	note
1	neg	neg	0.84299165	pos	0.15700835	
2	neg	neg	0.92962265	pos	0.07037741	
3	neg	neg	0.99856985	pos	0.0014301186	
4	neg	neg	0.9985342	pos	0.0014658204	
5	neg	neg	0.9579537	pos	0.04204625	
6	neg	neg	0.94263494	pos	0.05736511	
7	neg	neg	0.821507	pos	0.1784931	
8	neg	neg	0.81683284	pos	0.18316716	
9	neg	pos	0.5701527	neg	0.4298473	ARTIFACT
10	neg	neg	0.63421845	pos	0.36578155	
11	neg	pos	0.5930605	neg	0.40693948	ARTIFACT
12	neg	neg	0.7828923	pos	0.21710768	
13	neg	neg	0.8812124	pos	0.11878755	
14	neg	neg	0.9986255	pos	0.0013745388	
15	neg	neg	0.93378127	pos	0.06621876	
16	neg	neg	0.8159753	pos	0.18402475	
17	neg	neg	0.8454006	pos	0.15459946	
18	neg	neg	0.9983199	pos	0.0016800314	
19	neg	neg	0.9445048	pos	0.055495206	
20	neg	neg	0.89692175	pos	0.103078246	
21	neg	neg	0.9177396	pos	0.08226045	
22	neg	neg	0.8821661	pos	0.11783392	
23	neg	neg	0.9985135	pos	0.0014864398	
24	neg	neg	0.8814524	pos	0.11854758	
25	neg	neg	0.94433916	pos	0.05566086	
26	neg	neg	0.95333856	pos	0.046661437	
27	neg	neg	0.9430197	pos	0.056980345	
28	neg	neg	0.90476465	pos	0.09523535	
29	neg	neg	0.8999137	pos	0.10008629	
30	neg	neg	0.9690318	pos	0.030968145	
31	neg	neg	0.84167653	pos	0.15832348	
32	neg	neg	0.81136423	pos	0.18863574	
33	neg	neg	0.733128	pos	0.266872	
34	neg	neg	0.89913976	pos	0.100860246	
35	neg	neg	0.92988354	pos	0.07011646	
