# Supplementary material for: A Weakly Supervised Approach for HPV Status Prediction in Oropharyngeal Carcinoma from H&E-Stained Slides
Source: Cancers (Basel). 2025 Dec 9;17(24):3938. doi: 10.3390/cancers17243938 (PMC12730384; doi:10.3390/cancers17243938)
Supplement: Supplementary file 1 [file cancers-17-03938-s001.zip › Supplementary File S4.pdf]

Supplementary File S4

Table S4: **Comparison of model strategies across the four studies.**

Study	Model Strategy
**Our study (CLAM)**	Weakly supervised CLAM architecture using ResNet-50 feature extraction; attention-based slide-level classification; 41-feature cell-level morphometric analysis with Random Forest for biological validation.
**Klein et al. 2023 (OPSCCnet)**	Two-stage pipeline: (1) semantic segmentation of viable tumor using a Feature Pyramid Network (FPN) with ResNet-18 encoder; (2) tile-level classification with a second ResNet-18 network; generation of a combined score based on tile-class prevalence and variance for prognostic stratification.
**Adachi et al. 2024 (Annot-CLAM)**	Weakly supervised CLAM extended with manually annotated tumor ROIs (Annot-CLAM); multi-magnification feature extraction; interpretability enhanced using CycleGAN image-to-image translation to visualize discriminative morphological patterns.
**Wang et al. 2023 (Digital-HPV score)**	Multiple-instance learning pipeline with customized triplet-ranking loss; weakly supervised patch scoring; top-k patch aggregation; integration of tumor microenvironment profiling through spatial tissue classification and immune cell quantification.

Table S5: **Dataset characteristics across the four studies.**

Study	Dataset Description
**Our study**	123 WSIs total (OPSCC-UNINA, n=113; TCGA-HNSC, n=10). External test set: 35 HPV-negative WSIs.
**Klein et al. 2023**	906 patients across four European centers + TCGA. Training cohort: 267 patients. Includes primary tumors and lymph node metastases.
**Adachi et al. 2024**	114 OPSCC biopsy WSIs (50 p16-positive, 64 p16-negative). Tumor ROIs manually annotated by expert pathologists.
**Wang et al. 2023**	412 TCGA-HNSC cases + 69 OPSCC cases from Sheffield. Multiple anatomical sub-sites included; combined histology, immune and spatial profiling.

Table S6: **Ground truth definitions used in the four studies.**

Study	Ground Truth Definition
**Our study**	HPV status defined primarily by p16 IHC; INNO-LiPA or ISH results available for subsets.
**Klein et al. 2023**	Dichotomous HPV-status identification using both p16 IHC and HPV-DNA detection (ISH or PCR).
**Adachi et al. 2024**	Binary classification based solely on p16 IHC positivity.
**Wang et al. 2023**	HPV infection status obtained from molecular assays (p16, DNA or RNA ISH, depending on TCGA documentation).

Table S7: **Model performance comparison across all studies.**

Study	Internal Performance	External Performance
**Our study**	Mean CV test AUC: 0.5324; mean test accuracy: 56.5%. Validation AUC: 0.7178.	External independent test (n=35 HPV-negative): 94.3% accuracy.
**Klein et al. 2023**	Train AUROC: 0.93. Test AUROC: 0.83 (n=639).	Using variance-filtering: AUROC 0.88; robust prognostic stratification across sites.
**Adachi et al. 2024**	Whole-tissue CLAM AUC: 0.802–0.834. Annot-CLAM AUC: up to 0.905.	External TCGA-HNSC (n=17): AUC 0.874; accuracy 0.824; F1 score 0.889.
**Wang et al. 2023**	Internal cross-validation AUROC: 0.9223.	External validation AUROC: 0.8371–0.8397 across Sheffield vs TCGA.

Table S8: **Summary of strengths and limitations.**

Study	Strengths	Limitations
**Our study**	Strong external specificity; interpretable attention mapping; integrated cell-level morphology validation.	Small dataset; p16-only GT introduces label noise; variable CV performance.
**Klein et al. 2023**	Largest multi-center dataset; robust segmentation + classification; excellent prognostic performance.	Complex two-part pipeline; depends on tumor segmentation quality.
**Adachi et al. 2024**	Annot-CLAM achieves high accuracy; interpretable via CycleGAN; strong generalization to TCGA.	Requires manual tumor annotations; small biopsy-based dataset.
**Wang et al. 2023**	Highest AUC overall; generalizes well across cohorts; integrates immune and spatial TME features.	Computationally intensive; combines diverse HNSCC sub-sites.
